# Reverse Total Shoulder Arthroplasty for a Rare Pathological Fracture of the Proximal Humerus Caused by Marginal Zone Lymphoma: A Case Report

**DOI:** 10.1002/ccr3.72868

**Published:** 2026-06-23

**Authors:** Fuhang Shuang, Huaixu Song, Jianbang Hu, Shaoran Fang, Shuai Guan, Guanghong Pu, Weijie Xie, Genbi Jiang, Lei Zhang, Junlin Wu, Tingdong Yang

**Affiliations:** ^1^ Department of Traumatic Surgery Northeast Yunnan Central Hospital Zhaotong China

**Keywords:** marginal zone lymphoma, pathological fracture, proximal humerus, reverse total shoulder arthroplasty, shoulder function

## Abstract

We report a rare pathological proximal humerus fracture caused by marginal zone lymphoma. The patient underwent reverse total shoulder arthroplasty followed by systemic therapy. At 15‐month follow‐up, functional recovery was excellent with no recurrence. Low‐energy fractures without trauma history warrant suspicion of underlying pathology.

## Key Clinical Message

Pathological fractures of the proximal humerus caused by marginal zone lymphoma (MZL) are extremely rare. Reverse total shoulder arthroplasty (RSA) offers superior functional outcomes compared with hemiarthroplasty in cases with compromised rotator cuff function. Multidisciplinary collaboration and postoperative systemic therapy are essential for optimal oncologic and functional outcomes.

## Graphical abstract

A 69‐year‐old woman had a trauma‐free proximal humerus pathological fracture due to MZL. RSA was performed, followed by systemic therapy. At 15 months, function was excellent with no recurrence. RSA is superior for cuff‐insufficient oncologic reconstruction. Multidisciplinary care optimizes outcomes.

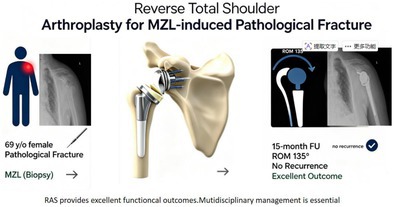

## 1 Introduction

The mechanization of agriculture has increased the incidence of high‐energy trauma, but low‐energy fractures in elderly patients should raise suspicion for underlying pathology [1]. Marginal zone lymphoma (MZL) is an indolent B‐cell non‐Hodgkin lymphoma, accounting for approximately 8% of all lymphomas [2]. Skeletal involvement in non‐Hodgkin lymphoma occurs in 3%–5% of cases, but MZL with bone involvement is exceedingly rare [3]. The proximal humerus is the third most common site for malignant bone tumors, yet pathological fractures due to MZL are rarely reported [4]. Importantly, such pathological fractures often mimic osteoporotic fractures and can be easily misdiagnosed, especially in elderly patients without obvious trauma [1].

Surgical options for proximal humerus reconstruction after tumor resection include hemiarthroplasty, reverse total shoulder arthroplasty (RSA), and allograft‐prosthetic composites [5]. RSA provides biomechanical advantages by medializing the center of rotation, enabling deltoid‐driven elevation even in the absence of a functional rotator cuff [6]. This makes RSA particularly suitable for oncologic reconstruction where cuff resection is inevitable [7]. We present a case of MZL‐induced pathological fracture of the proximal humerus managed successfully with RSA and systemic therapy.

## 2 Case Report

### 2.1 History and Examination

A 69‐year‐old female presented with left shoulder pain and limited motion for 7 days without any history of trauma. She reported waking up with pain and difficulty moving the shoulder. Physical examination revealed swelling and tenderness over the proximal humerus, with palpable crepitus. Active and passive range of motion was severely restricted. Multiple palpable lymph nodes were noted in the left axillary and cervical regions. Neurovascular examination was normal. Routine blood tests were unremarkable.

### 2.2 Imaging and Biopsy

**FIGURE 1:**
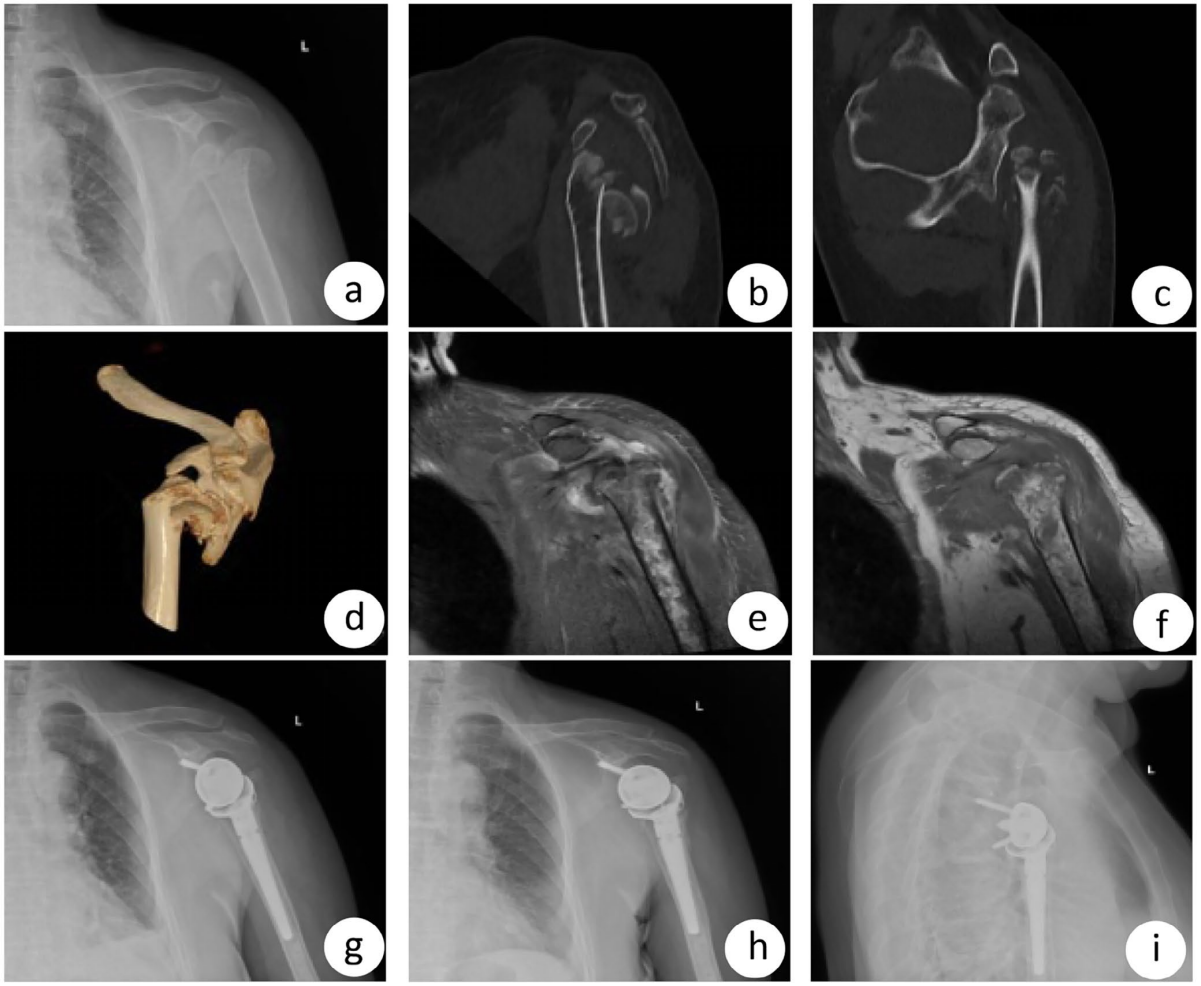
(a) Preoperative radiograph showing a comminuted fracture of the proximal humerus with bone destruction. (b–d) Preoperative CT scans showing a comminuted fracture of the proximal humerus with multiple abnormal signals in the medullary cavity. (e, f) Preoperative MRI revealing a comminuted fracture of the proximal humerus with rotator cuff injury and multifocal abnormal signals in the medullary cavity, suggestive of a marrow‐based lesion. (g) Postoperative radiograph showing satisfactory prosthesis positioning. (h, i) Radiographs at 15 months postoperatively showing the prosthesis remains well positioned with no significant changes compared to (g).

Radiographs showed a comminuted fracture of the proximal humerus with significant osteopenia (Figure 1a). CT revealed bone destruction and abnormal signals in the medullary cavity (Figure 1b–d). MRI demonstrated a comminuted fracture with suspected partial injuries of the supraspinatus and infraspinatus tendons, and multifocal cystic lesions suggestive of marrow‐based pathology (Figure 1e,f). Intraoperative findings later confirmed partial tears of both tendons. Ultrasound‐guided biopsy was performed targeting the intramedullary osteolytic lesion of the proximal humerus, which revealed diffuse infiltration of small lymphocytes. Immunohistochemistry confirmed the diagnosis of marginal zone lymphoma: CD20(+), CD79a(+), CD10(−), Bcl‐2(+), Bcl‐6(−), CD23(−), CyclinD1(−), CD3(scattered+), CD5(scattered+), CD43(−), LEF‐1(−), CD21(+), MPO(tumor cells−), MUM1(−), MYC(−), Ki‐67(≈10%+).

### 2.3 Multidisciplinary Assessment

A multidisciplinary team including orthopedics, hematology, and oncology confirmed the diagnosis of primary MZL of bone. No contraindications to surgery were identified.

### 2.4 Surgical Procedure

Under general anesthesia, the patient was placed in the beach‐chair position. A deltopectoral approach was used. The cephalic vein was preserved. The proximal humerus was found to be comminuted with soft tissue involvement. The rotator cuff tendons were tagged with high‐tension sutures. Given that MZL is an indolent lymphoma, we performed thorough debridement of the intramedullary lytic and tumor‐involved bone tissue, while preserving viable bone fragments for soft tissue attachment and reconstruction. The humeral head fragments were removed. The glenoid was exposed and prepared for placement of a glenoid baseplate. After canal preparation and cementing, a reverse total shoulder prosthesis was implanted. The rotator cuff tendons, including supraspinatus, subscapularis, teres minor, and the partially torn infraspinatus, were debrided and anatomically repaired and reattached to the prosthesis. Intraoperative fluoroscopy confirmed proper positioning of the prosthesis. Blood loss was 400 mL; no transfusion was required.

### 2.5 Postoperative Course

Postoperative radiographs showed satisfactory prosthesis position (Figure 1g). The patient wore a shoulder brace for 6 weeks. A structured rehabilitation protocol was initiated: pendulum exercises and passive motion within 1 week, progressing to active motion by 4–12 weeks.

At 2 weeks post‐surgery, the patient was referred to hematology for systemic therapy. She completed 6 cycles of chemotherapy and immunotherapy. Post‐treatment bone marrow biopsy showed significant remission with no evidence of lymphoma.

### 2.6 Follow‐up and Outcomes

At 3 months, the patient achieved active abduction of 110° (measured with elbow flexed 90° and arm adducted). At 15 months, range of motion was as follows: abduction 135°, adduction 45°, forward flexion 125°, extension 50°, internal rotation (measured with elbow flexed 90° and arm adducted) 90°, external rotation 80°. Radiographs showed stable implants with no evidence of loosening, dislocation, or tumor recurrence (Figure 1h,i). The patient was highly satisfied with the functional outcome.

## 3 Discussion

Pathological fractures of the proximal humerus due to MZL are exceptionally rare [3]. MZL is an indolent lymphoma, and skeletal involvement is often asymptomatic or misdiagnosed as osteoporotic fracture [1]. In a pathological study of 1642 amputation and 4163 arthroplasty specimens, only 5 cases of MZL were incidentally detected, yielding a detection rate of 0.08% [8]. In this case, the absence of trauma history and the imaging findings prompted biopsy, which was critical for diagnosis [4].

Reconstruction options after proximal humerus tumor resection include hemiarthroplasty, RSA, and allograft‐prosthetic composites [5]. Hemiarthroplasty relies on an intact rotator cuff and tuberosity healing, which are often compromised in tumor surgery [9]. RSA, by contrast, provides stability and active motion through the deltoid muscle, even in the absence of cuff function [7]. RSA medializes the center of rotation, enabling deltoid‐driven elevation, which is especially valuable in oncologic cases. Recent studies and Musculoskeletal Tumor Society guidelines recommend RSA over hemiarthroplasty for proximal humerus reconstruction to reduce instability, improve function, and lower reoperation rates [10].

Since marginal zone lymphoma was preoperatively confirmed as an indolent tumor [11], a multidisciplinary discussion with hematology and oncology was performed. The team confirmed that postoperative radiotherapy yields excellent local tumor control for indolent lymphomas with bone involvement. Therefore, we determined that radical resection of all bone fragments was not necessary for this indolent disease. Adequate debridement of lytic and tumor‐involved bone tissue, followed by standardized postoperative radiotherapy, is sufficient to achieve satisfactory local control [3]. This approach preserves supporting bone for soft tissue repair and implant stability without compromising oncologic safety and is consistent with clinical principles for managing indolent bone lymphomas [12].

Although complication rates including dislocation and reoperation are relatively higher in oncologic RSA than in non‐oncologic settings [12], careful rotator cuff reconstruction, deltoid preservation, and high‐tension suture repair enhance joint stability [9]. The indolent nature of MZL, limited bone resection, and multidisciplinary management further minimized complications in this case. Postoperative systemic therapy is essential to control disease progression and ensure long‐term implant survival. Individualized reconstruction and multidisciplinary collaboration remain crucial for optimal outcomes.

## 4 Conclusion

Marginal zone lymphoma of the proximal humerus presenting as a pathological fracture is rare and may mimic osteoporotic fractures. A high index of suspicion is warranted in low‐energy fractures without trauma history. Reverse total shoulder arthroplasty provides excellent functional outcomes and is preferred over hemiarthroplasty when rotator cuff function is compromised. Systemic therapy is essential to prevent recurrence. Multidisciplinary collaboration is key to successful management.

## Author Contributions

**Shaoran Fang:** supervision. **Huaixu Song:** resources. **Guanghong Pu:** data curation. **Genbi Jiang:** data curation. **Junlin Wu:** data curation. **Lei Zhang:** data curation. **Jianbang Hu:** funding acquisition. **Shuai Guan:** data curation. **Fuhang Shuang:** writing – original draft, writing – review and editing. **Weijie Xie:** data curation. **Tingdong Yang:** data curation.

## Funding

Fund Projects: Yunnan Provincial Department of Education (2025J0352); Clinical Research Project of Northeastern Yunnan Central Hospital (2025ZXYY008).

## Consent

Written informed consent was obtained from the patient for publication of this case report and accompanying images.

## Conflicts of Interest

The authors declare no conflicts of interest.

## Data Availability

The data that support the findings of this study are available from the corresponding author upon reasonable request.
